# Oil from transgenic *Camelina sativa* as a source of EPA and DHA in feed for European sea bass (*Dicentrarchus labrax* L.)

**DOI:** 10.1016/j.aquaculture.2020.735759

**Published:** 2021-01-15

**Authors:** M.B. Betancor, A. MacEwan, M. Sprague, X. Gong, D. Montero, L. Han, J.A. Napier, F. Norambuena, M. Izquierdo, D.R. Tocher

**Affiliations:** a Institute of Aquaculture, Faculty of Natural Sciences, University of Stirling, Stirling FK9 4LA, United Kingdom; b Grupo de Investigación en Acuicultura (GIA), Instituto Universitario Ecoaqua, Universidad de Las Palmas de Gran Canaria, Ctra. Taliarte s/n, 35214 Telde, Las Palmas, Canary Islands, Spain; c Department of Biological Chemistry and Crop Protection, Rothamsted Research, Harpenden AL5 2JQ, United Kingdom; d Biomar AS, Havnegata 9, Pirsenteret 3, Trondheim 7010, Norway

**Keywords:** EPA, DHA, Camelina, Seabass, Fish oil

## Abstract

Aquaculture, the fastest growing food production sector cannot continue to rely on finite stocks of marine fish as the primary source of the omega-3 (n-3) long-chain polyunsaturated fatty acids (LC-PUFA), eicosapentaenoic acid (EPA; 20:5n3) and docosahexaenoic acid (DHA; 22:6n-3), for feeds. A four-month feeding trial was conducted to investigate the impact of a *de novo* oil, with high levels of EPA and DHA, obtained from transgenic *Camelina sativa* on growth performance, tissue fatty acid profiles, and expression of lipid metabolism genes when used as a replacement for fish oil in feed for European seabass (*Dicentrachus labrax*). Triplicate groups of 50 juvenile fish (initial weight 16.7 ± 0.92 g) per tank were fed for 4 months with one of three isolipidic and isoproteic experimental diets consisting of a standard diet containing a commercial blend of fish oil and rapeseed oil (CFO), a diet containing transgenic *Camelina* oil (TCO), or a blend of fish oil and rapeseed oil with enhanced levels of EPA and DHA (EFO) formulated to match the n-3 LC-PUFA profile of the TCO feed. Final weight of fish fed the GM-derived oil was not different to fish fed either CFO or EFO. Slight lower growth performance of fish fed TCO at the beginning of the trial was related to transient reduced feed intake, possibly caused by glucosinolates in the raw *Camelina sativa* oil. The GM-derived oil improved the nutritional quality of the fish fillet by enhancing total n-3 PUFA levels compared to the fish fed the other two feeds, and maintained flesh EPA and DHA at the same levels as in fish fed the diets containing fish oil. The metabolic response in liver and intestine was generally relatively mild although diets TCO and EFO seemed to trigger a metabolic response consisting of an up-regulation of both β-oxidation (*cpt1a*) and fatty acid transport (*fabp1*), possibly reflecting higher levels of LC-PUFA. Overall, the present study indicated that an oil of terrestrial origin, *Camelina sativa*, when engineered to contain high levels of EPA and DHA can replace fish oil in feeds for European seabass with no detrimental impact on growth or feed efficiency, while also maintaining or increasing tissue n-3 LC-PUFA contents.

## Highlights

• An oil containing EPA and DHA from GM-*Camelina sativa* was tested in seabass.
• Growth and feed efficiency were similar in fish fed fish oil and the GM-derived oil.
• The GM-derived oil enhanced total n-3 PUFA level in fish fillet.
• Oil from GM-*Camelina sativa* can effectively replace fish oil in feeds for seabass.

## 1 Introduction

Fish are the primary dietary source of n-3 LC-PUFA for humans, where eicosapentaenoate (EPA; 20:5n-3) and docosahexaenoate (DHA; 22:6n-3) in particular have been recognized to provide health benefits (Calder, 2018; Shahidi and Ambigaipalan, 2018; Innes and Calder, 2020). However, LC-PUFA are also essential nutrients for many fish species (GOED, 2020; Tocher et al., 2019). In vertebrates, EPA, DHA, and arachidonic acid (ARA; 20:4n-6) can be synthesized by the sequential desaturation and elongation of *α*-linolenic acid (ALA; 18:3n-3) and linoleic acid (LA; 18:2n-6), respectively (Salem et al., 1996; Sprecher et al., 1995). However, the rate of biosynthesis is usually very low and so *in vivo* production of EPA and DHA is limited in most vertebrates (Tocher et al., 2019). The primary producers of n-3 LC-PUFA are microalgae that are able to directly synthesize EPA and DHA, which are then accumulated up the marine food chain to fish through the consumption of phytoplankton and zooplankton (Harris, 2010). For this reason, fish and seafood are the primary sources of n-3 LC-PUFA in the human diet (GOED, 2020). A daily dose of 500 mg of EPA + DHA is recommended for optimal health in humans, which requires consuming at least two portions of fish per week (GOED, 2020).

Farmed fish also require EPA and DHA supplied in the diet to ensure adequate growth and health, but also to accumulate n-3 LC-PUFA in order to pass on to human consumers. Traditionally this was ensured by including the marine ingredients, fishmeal (FM) and fish oil (FO), in feeds. However, FM and FO are finite and limited resources, which limits the supply of EPA and DHA to aquaculture (Tocher et al., 2019). Thus, as the main user and provider of n-3 LC-PUFA, the aquaculture industry has been a central driver in research aiming to alleviate the global shortfall in these key nutrients (Betancor et al., 2017; Tocher et al., 2019). Thus, as the industry grows and develops more sustainable practices, marine ingredients have been increasingly replaced in feeds by increasing proportions of terrestrial plant-based ingredients that lack LC-PUFA (Tocher, 2015).

While finding viable alternatives using new, *de novo* sources of LC-PUFA with appropriate inclusion of DHA and EPA is inherently complex, it is also highly necessary. Alternative lipid sources of marine origin can offer comparable nutrient profiles to FO, however most fisheries are already operating at or above sustainable harvest limits (FAO, 2018). Biotechnological initiatives to mass produce microalgae or zooplankton (krill and copepods) have been suggested as sustainable alternatives, but the actuality remains restricted largely by technological and (predominantly) economic challenges (Sprague et al., 2017; Tocher et al., 2019; Fabris et al., 2020). Incorporation of alternatives such as vegetable and rendered oils into feeds has led to dietary dilution of n-3 LC-PUFA, as well as an increase in the amount of n-6 PUFA (Glencross, 2009; Sprague et al., 2016).

Metabolic engineering of oilseed crops, specifically *Camelina sativa* or false flax is a feasible solution to meet n-3 LC-PUFA requirements in replacing fish oil (Haslam et al., 2015). Production of *C. sativa* containing n-3 LC-PUFA was accomplished by inserting seven fatty acyl desaturase and elongase genes from various marine microalgae species, resulting in genetically-modified (GM) *Camelina* plants capable of producing EPA and DHA (Ruiz-Lopez et al., 2014). Trials using GM *Camelina* in Atlantic salmon (*Salmo salar*) and gilthead sea bream (*Sparus aurata*) as a replacement for FO have shown no negative effects on growth performance, while enhancing accumulation and deposition of n-3 LC-PUFA compared to non-GM vegetable oil alternatives (Betancor et al., 2015a, Betancor et al., 2015b, Betancor et al., 2016a, Betancor et al., 2016b, Betancor et al., 2017, Betancor et al., 2018). Such findings are promising as they demonstrate the success of GM *Camelina* in restoring EPA and DHA in farmed fish to the levels found prior to the application of sustainable feeds (Sprague et al., 2016; Tocher et al., 2019).

The present study aimed to evaluate the effectiveness of oil from GM *Camelina sativa* as a dietary source of EPA and DHA in feeds for European seabass (*Dicentrarchus labrax*), a non-oily marine teleost species with limited LC-PUFA biosynthesis capacity. The trial compared sea bass fed the transgenic Camelina oil (TCO; EPA + DHA at 15.5% total fatty acids) with fish fed a commercial feed formulation containing FO and rapeseed oil (CFO; EPA + DHA at 11.7% total fatty acids), and an enhanced fish and rapeseed oil blend (EFO; EPA + DHA at 14.2% total fatty acids). Specific objectives were to evaluate the effects of transgenic Camelina oil in feeds on growth performance, feed efficiency, tissue fatty acid profiles and the expression of lipid-related genes in liver.

## 2 Materials and methods

### 2.1 Diets and feeding trial

Three isolipidic and isoproteic diets were formulated with different lipid sources and, therefore, different fatty acid profiles (Table 1). Diets were manufactured at the BioMar Tech Centre (Brande, Denmark) by vacuum coating identical dry basal extruded pellets with either FO and rapeseed oil in a commercial-like blend (CFO), transgenic *Camelina* oil (TCO; EPA 8.2%, DPA 5.7%, DHA 7.4% of total fatty acids), or an enhanced FO and rapeseed oil blend (EFO) to provide similar EPA and DHA levels to the TCO diet (Table 1). Details about the production of the GM-derived oil have been described previously (Ruiz-Lopez et al., 2014). A total of 450 juvenile European seabass with an average initial body weight of 16.7 ± 0.9 g (mean ± SD) were distributed into nine seawater tanks (50 fish per tank) and fed one of three experimental diets in triplicate for 125 days (approximately 4 months). The experimental setup used 500 L tanks supplied with flow-through seawater at ambient temperature (20 ± 1 °C) and a salinity of 34 g L^−1^. All fish were fed until apparent satiation, with feed intake (FI) determined as the difference between the amount of feed provided minus the amount of uneaten feed that was collected daily. All procedures were conducted in accordance with the regulations set forward by the Spanish RD 53/2013 (BOE 8th February 2013) and Directive 2010/63/EU of the European Parliament, and of the Council of 22 September 2010 on the protection of animals used for scientific purposes. Additionally, the experimental protocol was approved both by the Bioethical Committee of the University of Las Palmas de Gran Canaria (DGG.364.OEBA) and the Animal Welfare and Ethical Review Board at the University of Stirling (AWERB/1617/238).

**Table 1:** Formulations (%) of the experimental feeds.

	CFO	TCO	EFO
Feed ingredients (%)			
Fish meal, BioMar A/S	25.0	25.0	25.0
Soya, non-GM SPC	7.5	7.5	7.5
Rapeseed meal	5.0	5.0	5.0
Sunflower meal	5.9	5.7	5.7
Wheat gluten	2.0	2.0	2.0
Maize gluten	15.1	15.2	15.2
Guar meal	7.5	7.5	7.5
Wheat	16.3	16.1	16.1
Fish oil	5.7	–	7.6
Rapeseed oil	7.2	–	5.6
Camelina oil (transgenic)	–	13.6	–
Premix	2.4	2.4	2.4
Yttrium oxide	0.05	0.05	0.05
Analyzed composition (%)			
Dry matter	92.0	92.1	92.0
Crude protein	41.4	41.8	41.7
Crude lipid	17.5	18.6	17.7
Ash	7.6	7.6	7.6
Fatty acid composition (% fatty acids)			
Total saturated^1^	19.2	16.1	20.6
Total monoenes^2^	43.7	24.0	39.9
18:2n-6	15.0	21.6	14.4
18:3n-6	0.1	1.6	0.1
20:4n-6	0.5	2.2	0.6
Total n-6 PUFA^3^	15.9	27.4	15.5
18:3n-3	5.3	9.0	4.6
20:5n-3	6.5	8.2	8.1
22:5n-3	0.9	3.8	1.1
22:6n-3	5.2	7.3	6.1
Total n-3 PUFA^4^	19.5	32.1	21.9
Total n-3 LC-PUFA	12.9	21.0	15.7
EPA/DHA	1.2	1.1	1.3

CFO, commercial fish and vegetable oil feed; EFO, enhanced fish and vegetable oil feed; LC-PUFA, long-chain polyunsaturated fatty acid; TCO, transgenic Camelina oil feed.

1 Includes14:0, 15:0, 16:0, 18:0, 20:0, 22:0, 24:0.

2 Includes 16:1n-9, 16:1n-7, 17:1, 18:1n-9, 18:1n-7, 20:1n-11, 20:1n-9, 20:1n-7, 22;1n-11, 22:1n-9, 24:1n-9.

3 Includes 20:2n-6, 20:3n-6; 22:4n-6, 22:5n-6.

4 Includes 18:4n-3, 20:3n-3, 20:4n-3.

### 2.2 Sample collection

Fish were fed for 4 months, with weight and length of all individual fish measured monthly. All fish were humanely euthanized at the end of the trial with an overdose of anaesthetic (2-phenoxyethanol; 8 mL/L), with the last feed provided 48 h prior to culling. Two whole fish per tank were immediately frozen at −70 °C and later homogenized, pooled per tank (*n* = 3) and freeze-dried prior to analysis of proximate composition. Liver and anterior intestine were aseptically removed from an additional four fish per tank and stabilized immediately in RNAlater (Sigma, Poole, UK). The samples were then maintained at 4 °C overnight before being stored at −20 °C prior to RNA extraction. Samples of brain, liver, intestine, gill, head kidney and muscle were collected from a further two fish per tank, and were immediately frozen at −70 °C prior to lipid and fatty acid analyses. Additionally, blood from 3 fish per tank (*n* = 9) was collected *via* the caudal vein by 1 mL heparinized syringes fitted with 18 G needles, transferred to 1.5 mL tubes and centrifuged at 1000*g* for 20 min to allow red blood cells (RBC) to separate before the RBC pellet was quickly frozen at −70 °C prior to fatty acid analyses.

### 2.3 Calculations

Biometric parameters were calculated as follows: Specific growth rate (SGR) = 100 × (LnW_f_ − LnW_o_) × D^−1^, where W_o_ is the initial stocking weight of fish, W_f_ is the final sampling weight, and D is the number of days in the trial (D = 125). Feed conversion ratio (FCR) = (F/N/D)/(W_g_/D), where F is the total feed consumed, N is the number of fish, D is the number of days in the trial, and W_g_ is the weight gain (W_f_ − W_o_). Fulton's condition factor (k) = 100 × (W_f_/L^3^), where W_f_ is the final weight (g) and L is the total length (cm). Feed intake (FI; %BW day^−1^) = FI/BW_mean_ × 100, where FI (g/day) is the average feed intake per fish per day and BW_mean_ is the mean body weight, which was calculated as BW_mean_ (g) = (W_f_ + W_o_)/2. Hepatosomatic index (HSI) = (W_l_/W_f_) × 100, where W_l_ is the weight of the liver, and W_f_ is the final weight of fish. Visceral somatic index (VSI) = (W_v_/W_f_) × 100, where W_v_ is the weight of all viscera, and W_f_ is the final weight of fish. Fat-somatic index (FSI) = (W_fat_/W_f_) × 100, where W_fat_ is the weight of all fat, and W_f_ is the final weight of fish.

### 2.4 Proximate composition

Two whole fish per tank were ground, freeze-dried and prepared as pooled homogenates (*n* = 3) prior to analyses of proximate composition (AOAC, 2010). Crude protein was determined by the Kjeldalh method using nitrogen content of sample (N × 6.25) with automated analysis (Tecator Kjeltec Auto 1030 analyser, Foss, Warrington, UK). Crude lipid content was determined by extracting total lipid followed by gravimetric quantitation (Folch et al., 1957). Moisture content was determined following drying in an oven at 110 °C for 24 h (AOAC, 2010). Ash content was determined following incineration in a muffle furnace at 600 °C for 24 h, all as per standardized procedures.

### 2.5 Tissue lipid content and fatty acid composition

Total lipid contents of tissues were determined after lipid extraction by homogenization in chloroform/methanol (2:1, by vol.) using an Ultra-Turrax tissue disrupter (Fisher Scientific, Loughborough, UK) following which lipid content was determined gravimetrically (Folch et al., 1957). Total lipids were resuspended in chloroform/methanol (2:1) + 0.01% (*w*/*v*) BHT to a concentration of 10 mg/mL for subsequent analyses. Fatty acid methyl esters (FAME) of total lipid were prepared by acid-catalyzed transesterification at 50 °C for 16 h (Christie, 1993). Fatty acid compositions were determined by gas liquid chromatography using a Fisons GC-8160 (Thermo Scientific, Milan, Italy). Chromcard for Windows (version 2.01; Thermoquest Italia S.p.A., Milan, Italy) was used to collect and process data, and fatty acids identified using known standards and data obtained from literature (Ackman, 1980; Tocher and Harvie, 1988).

For RBC, fatty acid composition was determined by direct methylation method. Approximately 100 μL of defrosted RBC was spotted onto Whatman 903 blood collection cards (GE Healthcare Ltd., Forest Farm Industrial Estate, Cardiff, UK) and air dried. The RBC sample was detached with forceps and placed into a screw-cap vial containing 1 mL of methylating solution (1.25 M HCl/methanol) and then incubated in a hot block at 70 °C for 1 h. The vials were allowed to cool to room temperature before adding 2 mL of distilled water and 2 mL of saturated KCl solution and FAME extracted using 2 mL of isohexane + BHT followed by a second extraction using 2 mL of isohexane alone. FAME were separated and quantified by gas liquid chromatography as above.

### 2.6 RNA extraction and quantitative PCR (qPCR)

Samples of liver and anterior intestine from four fish per tank were homogenized in TriReagent® (Sigma-Aldrich, Dorset, UK) using a bead tissue disruptor (Bio Spec, Bartlesville, Oklahoma, USA). Total RNA was isolated using organic extraction, and quantity and quality determined by spectrophotometry using a Nanodrop ND-1000 (Labtech Int., East Sussex, UK) and electrophoresis using 200 ng of total RNA in a 1% agarose gel. RNA was pooled (6 μg of total RNA per fish) from two fish per tank for each tissue, thereby producing six pools (*n* = 6, 2 pools per tank) per dietary treatment with concentration of pools determined by spectrophotometry (Nanodrop). cDNA was synthesized using 2 μg of total RNA and random primers in 10 μL reactions. The resulting cDNA was diluted 20-fold with milliQ water.

Expression of genes of interest (Supplementary Table S1) was determined by quantitative PCR using a Biometra TOptical Thermocycler (Analytik Jena, Goettingen, Germany) using 96-well plates in duplicate 10 μL reaction volumes containing 1 μL of the primer (Table 3) corresponding to the analyzed gene (10 pmol/ μL), 1.5 μL of miliQ water, 5 μL SYBR Green Real-Time PCR Master Mix and 2.5 μL of cDNA, with the exception of the house-keeping genes, which were determined using 2 μL of cDNA. Negative controls (NTC, non template control and RT-, reverse transcriptase minus) containing no cDNA were also included. Standard amplification included a UDG (Uracil-DNA glycosylases) pre-treatment for 2 min at 50 °C, initial denaturation for 10 min at 95 °C, proceeded by 35 cycles: 15 s at 95 °C, 30 s at 60 °C, and 30 s at 72 °C. After which the melting curve was added (60–95 °C).

Results were normalized for liver and anterior intestine using housekeeping genes *ef1α* (M-values of 0.473 and 0.319, respectively), *bactin* (M-values of 0.473 and 0.319, respectively), and *rplp0* (M-values of 0.565 and 0.375, respectively) whose expression did not vary among treatments. The efficiency of the primers for each gene was evaluated by serial dilutions to ensure that it was close to 100%, quantified using a standard curve. Stability of housekeeping genes was determined independently for liver and anterior intestine samples using Genorm (Xie et al., 2012).

### 2.7 Statistical analysis

All data were presented as means ± standard deviation (SD; *n* = 3; *n* = 9 for RBC), except for the qPCR analysis that are shown as means ± standard error (SE; *n* = 6), with all statistical analyses performed using SPSS software (IBM SPSS Statistics 19; SPSS Inc., Chicago, IL, USA). Arcsine transformation was applied to percentage data to ensure binomial distribution. Differences between mean values were analyzed by *t*-test and one-way analysis of variance (ANOVA) followed, when pertinent, by a multiple comparison test (Tukey). Differences rejecting the null hypothesis were reported as statistically significant when *P* < .05. The Pfaffl method, which accounts for differences in efficiencies, was then used to calculate the relative gene expression (gene expression fold changes) among treatments (Pfaffl et al., 2002).

## 3 Results

### 3.1 Fish performance and proximate composition

All experimental diets were well accepted by the fish and all treatment groups increased in weight by approximately six-fold by the end of the four-month trial (Table 2). Survival was unaffected by dietary treatment (not shown). Although no significant differences were observed, fish fed the EFO diet had the highest final weight, final length and weight gain, but specific growth rate (SGR) was significantly higher in fish fed diet EFO compared to fish fed TCO, with fish fed CFO displaying intermediate values (Table 2). Feed intake and feed conversion ratio were not affected by diet, and no significant differences were observed in condition factor k, or hepatosomatic (HSI), viscerosomatic (VSI) and fat-somatic (FSI) indices.

**Table 2:** Growth performance, survival, feed utilization and whole body biochemical composition of European seabass after four months of feeding the experimental diets.

	CFO	TCO	EFO
Initial weight (g)	16.6 ± 1.0	16.7 ± 0.9	16.7 ± 0.9
Final weight (g)	100.8 ± 18.5	98.9 ± 16.9	107.0 ± 19.0
Initial length (cm)	10.5 ± 0.3	10.6 ± 0.4	10.5 ± 0.4
Final length (cm)	18.7 ± 1.2	18.7 ± 1.2	19.0 ± 1.1
Weight gain (g)	84.2 ± 2.9	82.2 ± 4.0	90.3 ± 3.5
HSI	2.0 ± 0.4	1.8 ± 0.4	1.8 ± 0.3
VSI	5.9 ± 1.7	5.5 ± 0.6	5.6 ± 1.0
FSI	6.5 ± 1.7	5.2 ± 0.9	7.8 ± 1.6
FI (kg/tank)	5.29 ± 0.13	4.99 ± 0.24	5.47 ± 0.22
FI (%BW day^−1^)	1.76 ± 0.05	1.68 ± 0.09	1.82 ± 0.04
FCR	1.3 ± 0.1	1.2 ± 0.1	1.2 ± 0.1
SGR	1.44 ± 0.35^ab^	1.42 ± 0.30^b^	1.50 ± 0.33^a^
*k*	1.4 ± 0.1	1.4 ± 0.1	1.3 ± 0.1

Whole body composition (% wet weight)			
Moisture	62.2 ± 0.3	63.3 ± 1.3	62.4 ± 1.2
Crude protein	24.3 ± 1.9	25.0 + 2.4	23.8 ± 1.7
Crude lipid	10.0 ± 0.8	8.7 ± 1.6	10.2 ± 1.2
Ash	3.5 ± 0.9	3.6 ± 0.7	3.1 ± 0.3

Results are means ± SD (*n* = 3). Different superscript letters within a row denote significant differences between diets as determined by one-way ANOVA with Tukey and Duncan's comparison test (*P* < .05). CFO, commercial fish and vegetable oil feed; EFO, enhanced fish and vegetable oil feed; FCR, feed conversion ratio; FSI, fat-somatic index; BW, body weight; HSI, hepatosomatic index; k, condition factor; SGR, specific growth rate; TCO, transgenic camelina oil feed; VSI, viscerosomatic index.

**Fig. 1:**
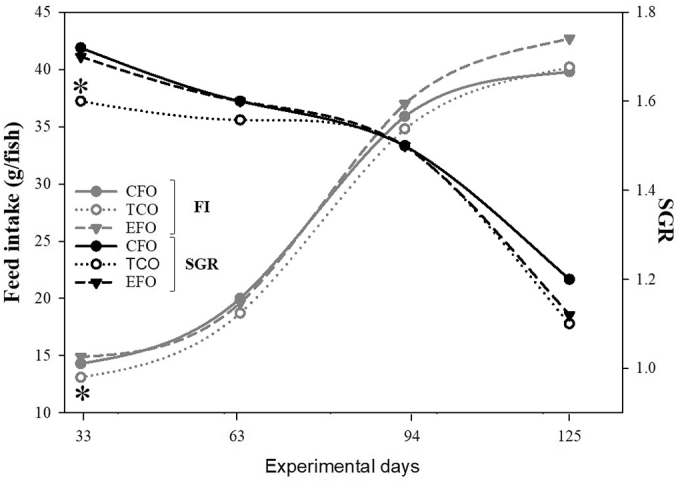
Effect of diet on feed intake (FI) and specific growth rate (SGR) throughout the nutritional trial. Depicted are the four sampling points at 33, 63, 94 and 125 days from the initiation of the feeding trial. The grey line corresponds to feed FI and the black line to SGR. Asterisks denote differences in the evaluated parameters as determined by one-way ANOVA. Diets contained either commercial fish oil (CFO), transgenic *Camelina sativa* oil (TCO), or fish oil at an enhanced DHA and EPA level (EFO).

When the performance data were split into the four different sampling points, fish fed CFO displayed the highest SGR and TCO the lowest, with EFO-fed fish showing intermediate values at the first sampling point (Fig. 1). Additionally, fish fed TCO showed the lowest feed intake (FI) at the first sampling point, whereas this parameter was not different among the experimental groups afterwards (Fig. 1). Diet had no effect on the biochemical composition of whole fish with no significant differences in protein, lipid, ash and moisture contents (Data not shown).

### 3.2 Tissue lipid contents and fatty acid compositions

#### 3.2.1 Liver

Lipid content of liver varied between 19.1 and 24.7% and, although there was a trend for higher lipid content in liver of sea bass fed diet EFO, this was not significant (*p* = .351; Table 3). Total n-3 PUFA and LC-PUFA were higher in liver of fish fed diet TCO with the differences being also significant for EPA, 20:4n-3 and ALA compared to fish fed either CFO or EFO (Table 3). Total n-6 PUFA was also highest in liver of fish fed TCO and, while all individual n-6 PUFA were higher in fish fed TCO, it was only significant with 18:2n-6, 18:3n-6, 20:2n-6 and ARA. Balancing these effects on PUFA, total monoenes were significantly lower in liver of sea bass fed diet TCO mainly due to a lower level of 18:1n-9, whereas there were no major significant effects on other saturated fatty acids.

**Table 3:** Total lipid content (% of wet weight) and total lipid fatty acid composition (% total fatty acids of liver of European seabass after 4 months of feeding the experimental diets.

	CFO	TCO	EFO
Lipid content	20.3 ± 5.2	19.1 ± 2.8	24.7 ± 5.1
14:0	2.1 ± 0.1	1.8 ± 0.2	2.0 ± 0.3
16:0	23.8 ± 2.8	21.4 ± 4.3	23.4 ± 0.7
18:0	5.6 ± 0.8	5.9 ± 1.4	7.0 ± 1.4
20:0	0.2 ± 0.0^b^	0.5 ± 0.1^a^	0.2 ± 0.0^b^
Total saturated^1^	32.1 ± 3.5	30.0 ± 5.2	32.9 ± 0.8
16:1n-7	4.8 ± 0.4	3.8 ± 0.7	4.4 ± 0.5
18:1n-9	40.8 ± 0.7^ab^	33.7 ± 4.9^b^	42.9 ± 2.4^a^
18:1n-7	2.9 ± 0.2^a^	2.3 ± 0.3^b^	2.9 ± 0.2^a^
20:1n-11	0.6 ± 0.7	0.1 ± 0.0	0.2 ± 0.0
20:1n-9	1.0 ± 0.8	2.5 ± 0.5	1.2 ± 0.2
20:1n-7	0.1 ± 0.1	0.1 ± 0.0	0.1 ± 0.0
22:1n-11	0.5 ± 0.0	0.4 ± 0.1	0.4 ± 0.1
22:1n-9	0.2 ± 0.0^b^	0.3 ± 0.1^a^	0.2 ± 0.0^b^
24:1n-9	0.3 ± 0.0	0.4 ± 0.1	0.3 ± 0.1
Total monoenes^2^	52.1 ± 0.5^a^	44.6 ± 4.2^b^	53.6 ± 1.6^a^
18:2n-6	5.9 ± 1.3^ab^	8.6 ± 2.7^a^	4.6 ± 0.8^b^
18:3n-6	0.4 ± 0.1^b^	1.1 ± 0.3^a^	0.4 ± 0.1^b^
20:2n-6	0.3 ± 0.1^b^	0.6 ± 0.2^a^	0.2 ± 0.1^b^
20:3n-6	0.1 ± 0.0	0.3 ± 0.1	0.0 ± 0.0
20:4n-6	0.4 ± 0.1^b^	1.3 ± 0.6^a^	0.3 ± 0.1^b^
22:5n-6	0.1 ± 0.0	0.0 ± 0.1	0.1 ± 0.0
Total n-6 PUFA^3^	7.0 ± 1.4^ab^	12.1 ± 3.4^a^	5.6 ± 1.0^b^
18:3n-3	1.6 ± 0.5^b^	3.0 ± 0.5^a^	1.2 ± 0.2^b^
18:4n-3	0.5 ± 0.1	0.7 ± 0.1	0.5 ± 0.1
20:3n-3	0.1 ± 0.0	0.3 ± 0.1	n.d.
20:4n-3	0.1 ± 0.0^b^	0.6 ± 0.2^a^	0.1 ± 0.0^b^
20:5n-3	2.6 ± 0.6^b^	3.7 ± 0.9^a^	2.6 ± 0.5^b^
22:5n-3	0.4 ± 0.2^b^	1.7 ± 0.5^a^	0.4 ± 0.1^b^
22:6n-3	3.1 ± 0.6^b^	5.4 ± 1.3^a^	2.8 ± 0.3^b^
Total n-3 PUFA^4^	8.5 ± 2.0^b^	15.4 ± 3.6^a^	7.5 ± 1.2^b^
Total n-3 LC-PUFA	6.3 ± 1.4^b^	11.7 ± 3.0^a^	5.9 ± 0.9^b^

Results are means ± SEM (*n* = 3). Different superscript letters within a row denote significant differences between diets as determined by one-way ANOVA with Tukey and Duncan's comparison test (*P* < .05). CFO, commercial fish and vegetable oil feed; EFO, enhanced fish and vegetable oil feed; PUFA, polyunsaturated fatty acids; TCO, transgenic Camelina oil feed.

1 Contains 15:0, 22:0, 24:0.

2 Contains 16:1n-9.

3 Contains 20:3n-6, 22;4n-6.

4 Contains 20:3n-3.

#### 3.2.2 Muscle (flesh)

Lipid content of sea bass muscle (flesh) was relatively low at around 4% with no significant effect of diet (Table 4). Total n-3 PUFA were significantly higher in muscle of fish fed diet TCO with significant differences for 20:4n-3 and 22:5n-3, whereas there were no differences in muscle EPA and DHA or total n-3 LC-PUFA in fish fed the different diets (Table 4). Total n-6 PUFA was also highest in muscle of fish fed diet TCO with almost all n-6 PUFA being higher but especially 18:2n-6. As with liver, the increased PUFA in muscle was balanced by lower proportions of monoenes, particularly 18:1n-9 and 16:1n-7. Although percentages of 14:0 and 16:0 were lower in fish fed diet TCO, diet had no significant effect on total saturates in sea bass flesh.

**Table 4:** Lipid content (% of wet weight) and total lipid fatty acid composition (% total fatty acids of muscle (flesh) of European seabass after 4 months of feeding the experimental diets.

	CFO	TCO	EFO
Lipid content	3.7 ± 0.3	4.3 ± 0.9	3.8 ± 1.6
14:0	2.5 ± 0.1^a^	1.5 ± 0.2^b^	2.8 ± 0.5^a^
16:0	16.0 ± 0.6^a^	14.5 ± 0.3^b^	15.9 ± 0.5^a^
18:0	4.0 ± 0.5	4.3 ± 0.1	3.7 ± 0.4
Total saturated^1^	23.2 ± 1.0	21.4 ± 0.3	23.2 ± 0.5
16:1n-7	3.9 ± 0.1^a^	2.6 ± 0.2^b^	4.3 ± 0.4^a^
18:1n-9	31.6 ± 1.0^a^	22.1 ± 1.1^b^	29.7 ± 2.8^a^
18:1n-7	2.9 ± 0.0	2.2 ± 0.1	2.9 ± 0.2
20:1n-9	1.8 ± 0.0	3.5 ± 0.4	2.1 ± 0.3
22:1n-11	1.0 ± 0.1^a^	0.8 ± 0.1^b^	1.0 ± 0.1^a^
Total monoenes^2^	42.8 ± 0.8	32.8 ± 1.1	41.6 ± 3.2
18:2n-6	11.3 ± 0.5^b^	15.6 ± 0.8^a^	11.7 ± 0.7^b^
18:3n-6	0.2 ± 0.0	1.1 ± 0.2	0.3 ± 0.1
20:2n-6	0.4 ± 0.0	0.9 ± 0.1	0.5 ± 0.1
20:3n-6	0.1 ± 0.0^b^	0.4 ± 0.0^a^	0.1 ± 0.1^b^
20:4n-6	0.7 ± 0.0	1.9 ± 0.1	0.7 ± 0.3
22:5n-6	0.2 ± 0.0^a^	0.1 ± 0.0^b^	0.2 ± 0.0^a^
Total n-6 PUFA^3^	13.0 ± 0.5^b^	20.3 ± 1.1^a^	13.6 ± 1.2^b^
18:3n-3	3.6 ± 0.2	5.6 ± 0.4	3.7 ± 0.3
18:4n-3	0.8 ± 0.1	0.9 ± 0.1	0.9 ± 0.1
20:3n-3	0.1 ± 0.0^b^	0.5 ± 0.1^a^	0.1 ± 0.1^b^
20:4n-3	0.3 ± 0.0^b^	1.1 ± 0.1^a^	0.4 ± 0.1^b^
20:5n-3	6.3 ± 0.1	6.4 ± 0.2	6.7 ± 0.4
22:5n-3	0.8 ± 0.3^b^	2.6 ± 0.2^a^	1.2 ± 0.3^b^
22:6n-3	8.0 ± 0.7	7.9 ± 0.4	7.1 ± 1.5
Total n-3 PUFA^4^	20.1 ± 1.2^b^	25.1 ± 0.6^a^	20.4 ± 2.4^b^
Total n-3 LC-PUFA	15.7 ± 1.0	18.6 ± 0.5	15.8 ± 2.3

Results are means ± SEM (n = 3). Different superscript letters within a row denote significant differences between diets as determined by one-way ANOVA with Tukey and Duncan's comparison test (*P* < .05). CFO, commercial fish and vegetable oil feed; EFO, enhanced fish and vegetable oil feed; TCO, transgenic Camelina oil feed.

1 Total saturated includes 15:0, 20:0, 22:0 and 24:0.

2 Total monounsaturated includes 16:1n-9,17:1, 20:1n-11, 20:1n-7, 22:1n-9 and 24:1n-9.

3 Total n-6 PUFA includes 20:3n-6 and 22:4n-6.

4 Total n-3 PUFA includes 20:3n-3.

#### 3.2.3 Red blood cells

Fish fed the TCO diet showed the highest percentages of 18:3n-3, 20:4n-3 and 22:5n-3, but there were no significant differences in the relative proportions of the other n-3 fatty acids. Fish fed diet TCO displayed higher proportions of total and all individual n-6 PUFA, apart from 22:5n-6, than fish fed the other diets. In contrast, the percentages of total monoenes in red blood cells were significantly lower in fish fed the TCO diet compared to those fed the other diets, whereas saturated fatty acids were largely unaffected by diet.

#### 3.2.4 Brain

Lipid content of brain was on average around 10% but there were no significant differences due to diet (Table 6). Brain was characterized by a much higher proportion of DHA compared to the other tissues. However, there were no significant differences in the proportions of n-3 PUFA, LC-PUFA, or individual n-3 fatty acids in brain of fish fed the different diets. However, fish fed diet TCO had a higher proportion of ARA compared to fish fed the other diets. As with liver and muscle, total monoenes were significantly lower in brain of sea bass fed diet TCO compared to the other diets, whereas total saturates were unaffected by diet.

#### 3.2.5 Gills

The lipid content of gills varied between 7 and 8% with no significant differences due to diet (Table 6). The proportions of all n-3 fatty acids, as well as those of total n-3 PUFA and LC-PUFA, were significantly higher in gills of sea bass fed diet TCO compared to fish fed the other diets, albeit not different to that of EFO-fed fish in the case of 18:4n-3 and EPA and DHA (Table 5). Total n-6 PUFA was also highest in gill of fish fed TCO due to significantly higher proportions of all individual n-6 fatty acids other than 22:5n-6. As with the other tissues above, the higher proportions of PUFA in gills of fish fed diet TCO were balanced by significantly lower proportions of monoenes, while saturated fatty acids were not significantly affected by diet.

**Table 5:** Lipid contents (% of wet weight) and fatty acid compositions (% total fatty acids) of red blood cells of European seabass after 4 months of feeding the experimental diets.

	CFO	TCO	EFO
14:0	1.2 ± 0.3^a^	0.6 ± 0.1^b^	1.2 ± 0.4^a^
16:0	20.0 ± 1.8	19.7 ± 1.4	21.1 ± 0.8
18:0	13.2 ± 1.7	14.7 ± 1.6	13.9 ± 1.0
Total saturated^1^	35.5 ± 3.3	36.7 ± 2.6	37.2 ± 1.0
16:1n-7	1.8 ± 0.5^a^	1.0 ± 0.1^b^	1.9 ± 0.4^a^
18:1n-9	23.7 ± 4.2^a^	15.0 ± 1.6^b^	18.7 ± 6.2^ab^
18:1n-7	2.4 ± 0.3^a^	1.7 ± 0.1^b^	2.2 ± 0.6^ab^
20:1n-9	1.5 ± 0.2^b^	2.7 ± 0.3^a^	1.3 ± 0.2^b^
Total monounsaturated^2^	32.1 ± 5.3^a^	22.9 ± 2.0^b^	29.9 ± 2.1^a^
18:2n-6	7.1 ± 0.8^b^	8.3 ± 0.2^a^	5.9 ± 0.8^b^
18:3n-6	0.1 ± 0.0^b^	0.5 ± 0.1^a^	0.2 ± 0.0^b^
20:2n-6	0.4 ± 0.0^b^	0.8 ± 0.1^a^	0.4 ± 0.0^b^
20:3n-6	0.1 ± 0.0	0.3 ± 0.0	0.1 ± 0.1
20:4n-6	1.8 ± 0.5^b^	4.5 ± 0.4^a^	1.9 ± 0.1^b^
22:5n-6	0.2 ± 0.0^a^	0.1 ± 0.0^b^	0.2 ± 0.0^a^
Total n-6 PUFA^3^	9.9 ± 0.5^b^	14.7 ± 1.2^a^	8.9 ± 0.7^b^
18:3n-3	1.5 ± 0.3^b^	1.9 ± 0.5^a^	1.1 ± 0.3^b^
18:4n-3	0.3 ± 0.1	0.2 ± 0.1	0.3 ± 0.0
20:3n-3	n.d.	0.3 ± 0.0	0.4 ± 0.0
20:4n-3	0.1 ± 0.0^b^	0.5 ± 0.1^a^	0.2 ± 0.3^b^
20:5n-3	6.4 ± 1.3	5.9 ± 0.3	7.3 ± 0.5
22:5n-3	0.7 ± 0.2^b^	2.1 ± 0.2^a^	0.7 ± 0.1^b^
22:6n-3	12.6 ± 4.2	13.8 ± 2.2	13.0 ± 1.7
Total n-3 PUFA^4^	21.5 ± 5.5	24.8 ± 2.6	23.0 ± 2.4
Total n-3 LC-PUFA	19.7 ± 2.7	22.7 ± 2.6	21.7 ± 2.7

1 Total saturated includes 15:0, 20:0, 22:0 and 24:0.

2 Total monounsaturated includes 17:1, 20:1n-11, 20:1n-7, 22:1n-11, 22:1n-9, 24:1n-9.

3 Total n-6 includes 20:3n-6 and 22:4n-6.

4 Total n-3 PUFA includes 20:3n-3.

**Table 6:** Lipid contents (% of wet weight) and fatty acid compositions (% total fatty acids) of brain and gill of European seabass after 4 months of feeding the experimental diets.

	CFO	TCO	EFO
Brain			
Lipid content	10.8 ± 2.4	9.5 ± 0.5	11.2 ± 2.7
Total saturated	26.8 ± 2.3	26.6 ± 0.5	26.6 ± 1.4
Total monoenes	37.2 ± 3.9^a^	30.8 ± 0.5^b^	37.0 ± 1.6^a^
18:2n-6	5.7 ± 2.9	6.5 ± 0.8	6.0 ± 1.6
18:3n-6	0.1 ± 0.1	0.5 ± 0.1	0.1 ± 0.0
20:2n-6	0.3 ± 0.0	0.1 ± 0.0	0.4 ± 0.1
20:4n-6	1.6 ± 0.5^b^	3.1 ± 0.1^a^	1.7 ± 0.4^b^
22:5n-6	0.1 ± 0.1	0.1 ± 0.0	0.1 ± 0.0
Total n-6 PUFA^1^	8.1 ± 2.5	10.9 ± 0.9	8.5 ± 1.4
18:3n-3	1.8 ± 1.0	2.3 ± 0.3	1.8 ± 0.5
18:4n-3	0.4 ± 0.2	0.3 ± 0.0	0.5 ± 0.2
20:4n-3	0.2 ± 0.1	0.5 ± 0.0	0.2 ± 0.1
20:5n-3	4.2 ± 0.5	3.9 ± 0.2	4.7 ± 0.7
22:5n-3	0.9 ± 0.0	1.6 ± 0.1	1.0 ± 0.1
22:6n-3	16.0 ± 4.4	17.7 ± 0.4	15.1 ± 2.2
Total n-3 PUFA^2^	23.4 ± 2.7	26.5 ± 0.4	23.4 ± 0.6
Total n-3 LC-PUFA	21.3 ± 3.8	23.9 ± 0.2	21.1 ± 1.3

Gill			
Lipid content	8.3 ± 1.0	7.0 ± 0.2	7.4 ± 1.0
Total saturated	23.2 ± 1.1	20.8 ± 0.5	23.6 ± 0.3
Total monoenes	45.1 ± 0.8^a^	32.3 ± 0.9^b^	43.4 ± 0.6^a^
18:2n-6	11.5 ± 0.6^b^	16.5 ± 0.7^a^	10.9 ± 0.3^b^
18:3n-6	0.2 ± 0.0^b^	1.2 ± 0.1^a^	0.3 ± 0.0^b^
20:2n-6	0.5 ± 0.0^b^	1.1 ± 0.2^a^	0.5 ± 0.0^b^
20:4n-6	0.8 ± 0.1^b^	2.2 ± 0.2^a^	0.9 ± 0.1^b^
22:5n-6	0.2 ± 0.0	0.1 ± 0.0	0.2 ± 0.0
Total n-6 PUFA^1^	13.3 ± 0.6^b^	22.1 ± 0.7^a^	12.9 ± 0.4^b^
18:3n-3	3.6 ± 0.2^b^	5.8 ± 0.3^a^	3.2 ± 0.1^b^
18:4n-3	0.8 ± 0.1^b^	0.9 ± 0.0^a^	0.9 ± 0.0^ab^
20:4n-3	0.3 ± 0.0^c^	1.2 ± 0.1^a^	0.3 ± 0.0^b^
20:5n-3	5.2 ± 0.3^b^	5.9 ± 0.1^a^	6.1 ± 0.1^a^
22:5n-3	0.9 ± 0.1^c^	2.8 ± 0.1^a^	1.1 ± 0.1^b^
22:6n-3	6.5 ± 0.5^b^	7.3 ± 0.0^a^	6.9 ± 0.3^ab^
Total n-3 PUFA^2^	17.5 ± 0.9^b^	24.5 ± 0.6^a^	18.8 ± 0.4^b^
Total n-3 LC-PUFA	13.2 ± 0.7^c^	17.8 ± 0.3^a^	14.7 ± 0.4^b^

Results are means ± SEM (n = 3). Different superscript letters within a row denote significant differences between diets as determined by one-way ANOVA with Tukey comparison test (*P* < .05). CFO, commercial fish and vegetable oil feed; EFO, enhanced fish and vegetable oil feed; TCO, transgenic Camelina oil feed.

1 Total n-6 PUFA includes 20:3n-6 and 22:4n-6.

2 Total n-3 PUFA includes 20:3n-3.

**Table 7:** Lipid content (% of wet weight) and total lipid fatty acid composition (% total fatty acids) of anterior intestine and head kidney of European seabass after 4 months of feeding the experimental diets.

	CFO	TCO	EFO
Anterior intestine			
Lipid content	7.2 ± 0.8	5.6 ± 0.2	6.0 ± 0.9
Total saturated	23.3 ± 0.8^b^	22.6 ± 0.6^b^	25.5 ± 1.2^a^
Total monoenes	41.0 ± 0.5^a^	27.2 ± 1.7^c^	37.1 ± 1.2^b^
18:2n-6	12.4 ± 0.7^b^	15.9 ± 0.8^a^	10.6 ± 1.1^b^
18:3n-6	0.2 ± 0.0	1.1 ± 0.0	0.2 ± 0.0
20:2n-6	0.5 ± 0.1	1.1 ± 0.0	0.6 ± 0.0
20:4n-6	1.3 ± 0.1^b^	3.5 ± 0.3^a^	1.6 ± 0.2^b^
22:5n-6	0.2 ± 0.0	0.2 ± 0.0	0.3 ± 0.0
Total n-6 PUFA^1^	14.7 ± 0.6^b^	22.6 ± 1.0^a^	13.8 ± 0.8^b^
18:3n-3	3.8 ± 0.3^b^	5.3 ± 0.3^a^	3.0 ± 0.4^b^
18:4n-3	0.7 ± 0.1	0.8 ± 0.0	0.7 ± 0.1
20:4n-3	0.3 ± 0.1b	1.0 ± 0.0a	0.3 ± 0.0b
20:5n-3	6.0 ± 0.2^b^	6.4 ± 0.3^ab^	7.0 ± 0.2^a^
22:5n-3	0.9 ± 0.1^c^	2.7 ± 0.0^a^	1.1 ± 0.0^b^
22:6n-3	8.1 ± 0.5^b^	10.2 ± 0.4^a^	10.2 ± 1.0^a^
Total n-3 PUFA^2^	20.1 ± 0.3^c^	27.1 ± 0.8^a^	22.6 ± 0.8^b^
Total n-3 LC-PUFA	15.5 ± 0.5^c^	21.0 ± 0.6^a^	18.9 ± 1.2^b^

Head kidney			
Lipid content	13.3 ± 0.4^a^	9.7 ± 1.1^b^	8.1 ± 1.3^b^
Total saturated	24.2 ± 0.4	26.2 ± 2.9	28.9 ± 2.4
Total monoenes	47.0 ± 0.8^a^	36.0 ± 1.5^b^	46.9 ± 2.6^a^
18:2n-6	11.7 ± 0.4^b^	16.3 ± 0.8^a^	10.2 ± 0.9^b^
18:3n-6	0.2 ± 0.0^b^	1.0 ± 0.1^a^	0.2 ± 0.0^b^
20:2n-6	0.5 ± 0.0	1.1 ± 0.0	0.5 ± 0.1
20:4n-6	0.5 ± 0.1^b^	1.6 ± 0.1^a^	0.6 ± 0.2^b^
22:5n-6	0.1 ± 0.0	0.1 ± 0.1	0.1 ± 0.1
Total n-6 PUFA^1^	13.1 ± 0.5^b^	20.8 ± 1.1^a^	11.6 ± 1.3^b^
18:3n-3	3.5 ± 0.0^b^	4.9 ± 0.6^a^	2.5 ± 0.4^c^
18:4n-3	0.7 ± 0.0	0.6 ± 0.1	0.6 ± 0.1
20:4n-3	0.3 ± 0.0^b^	0.8 ± 0.2^a^	0.2 ± 0.0^b^
20:5n-3	4.5 ± 0.2	3.9 ± 0.9	3.9 ± 1.1
22:5n-3	0.7 ± 0.0^b^	1.8 ± 0.4^a^	0.6 ± 0.2^b^
22:6n-3	4.8 ± 0.3	4.0 ± 1.2	3.6 ± 1.5
Total n-3 PUFA^2^	14.8 ± 0.6	16.6 ± 3.4	11.6 ± 3.4
Total n-3 LC-PUFA	10.6 ± 0.5	11.1 ± 2.7	8.5 ± 2.9

Results are means ± SEM (*n* = 3). Different superscript letters within a row denote significant differences between diets as determined by one-way ANOVA with Tukey comparison test (*P* < .05). CFO, commercial fish and vegetable oil feed; EFO, enhanced fish and vegetable oil feed; TCO, transgenic Camelina oil feed.

1 Total n-6 PUFA includes 20:3n-6 and 22:4n-6.

2 Total n-3 PUFA includes 20:3n-3.

#### 3.2.6 Anterior intestine

The lipid content of anterior intestine of sea bass was not significantly affected by diet and varied between 5.6 and 7.2% (Table 7). The proportions of total n-3 PUFA and LC-PUFA, including EPA and DHA, were significantly higher in anterior intestine of fish fed diet TCO compared to fish fed the CFO diet, with levels in fish fed the EFO diet being generally intermediate (Table 7). Total n-6 PUFA was also highest in anterior intestine of fish fed TCO due to significantly higher proportions of both ARA and 18:2n-6. Monoenes in anterior intestine showed the opposite pattern to PUFA and, therefore, the proportion of monoenes was highest in fish fed diet TCO, lowest in fish fed CFO, and intermediate in fish fed EFO. The proportion of saturated fatty acids was higher in anterior intestine of sea bass fed diet EFO compared to fish fed the other two diets.

#### 3.2.7 Head kidney

The lipid content of head kidney was over 13% in fish fed CFO and was significantly lower (<10%) in sea bass fed the TCO and EFO diets (Table 7). Total n-3 PUFA and LC-PUFA were numerically higher in liver of fish fed diet TCO compared to fish fed the other feeds, but there were no significant differences (Table 7). Total n-6 PUFA was also highest in the head kidney of fish fed TCO due mainly to higher proportions of 18:2n-6 and ARA. Balancing these dietary effects on PUFA, total monoenes were significantly lower in head kidney of sea bass fed diet TCO compared to fish fed the other diets, whereas there was significant effect of diet on total saturates.

### 3.3 Liver and anterior intestine gene expression

#### 3.3.1 LC-PUFA biosynthetic genes

**Fig. 2:**
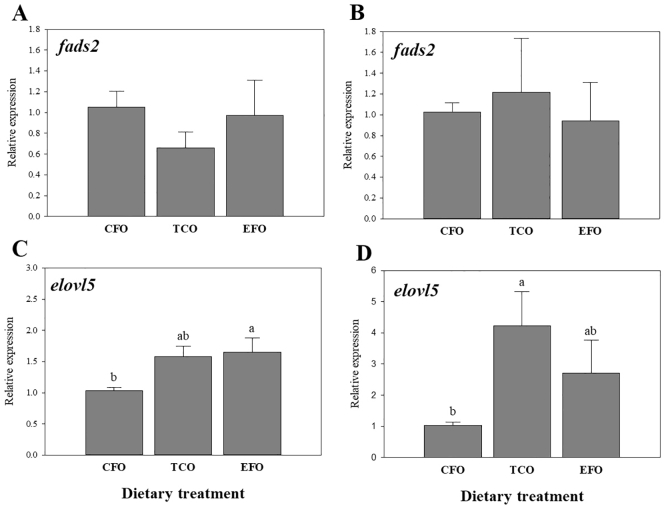
Expression of genes of long-chain polyunsaturated fatty acid (LC-PUFA) biosynthesis in (A and C) liver and (B and D) anterior intestine of European seabass as determined by qPCR. Results are normalized expression ratios (mean ± standard error; *n* = 6). Different superscript letters denote differences in expression as determined by one-way ANOVA (*p* < .05). Diets contained either commercial fish oil (CFO), transgenic *Camelina sativa* oil (TCO), or fish oil at an enhanced DHA and EPA level (EFO). *elovl5*, fatty acid elongase 5; *fads2*, fatty acyl desaturase 2.

No significant differences were found in fatty acid desaturase 2 (*fads2*) expression in liver (Fig. 2A) or anterior intestine (Fig. 2B). On the contrary, dietary regulation of fatty acid elongase 5 (*elovl5*) expression was observed in both the liver (Fig. 2C) and anterior intestine (Fig. 2D) being up-regulated in fish fed EFO and TCO, respectively.

#### 3.3.2 Lipid metabolism genes

**Fig. 3:**
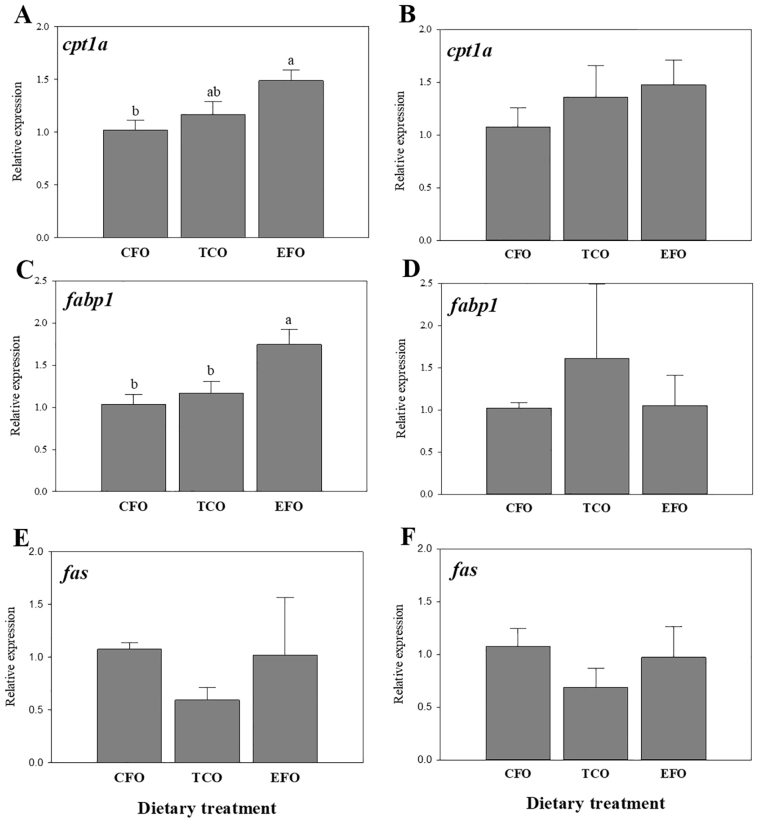
Expression of lipid metabolism genes in liver (A, C and E) and anterior intestine (B, D and F) of European seabass as determined by qPCR. Results are normalized expression ratios (mean ± standard error; n = 6). Different superscript letters denote differences in expression as determined by one-way ANOVA (*p* < .05). Diets contained either commercial fish oil (CFO), transgenic *Camelina sativa* oil (TCO), or fish oil at an enhanced DHA and EPA level (EFO). *cpt1a*, carnitine palmitoyltransferase 1A; *fapb1*, fatty acid binding protein 1; *fas*, fatty acid synthase.

The expression of the mitochondrial enzyme carnitine palmitoyltransferase 1A (*cpt1a*) was up-regulated in the liver of EFO-fed fish compared to fish fed the commercial standard diet (Fig. 3A). No significant differences were found in *cpt1a* expression in anterior intestine, although there was a similar non-significant trend to liver with EFO > TCO > CFO (Fig. 3B). The expression levels of fatty acid binding protein (*fabp1*) were also up-regulated in the liver of fish fed EFO (Fig. 3C), with no difference observed for the same gene in anterior intestine (Fig. 3D). There were no significant differences in expression of fatty acid synthase (*fas*) in liver (Fig. 3E) or anterior intestine (Fig. 3F), although expression was numerically lowest in fish fed TCO in both tissues.

#### 3.3.3 Transcription factors

**Fig. 4:**
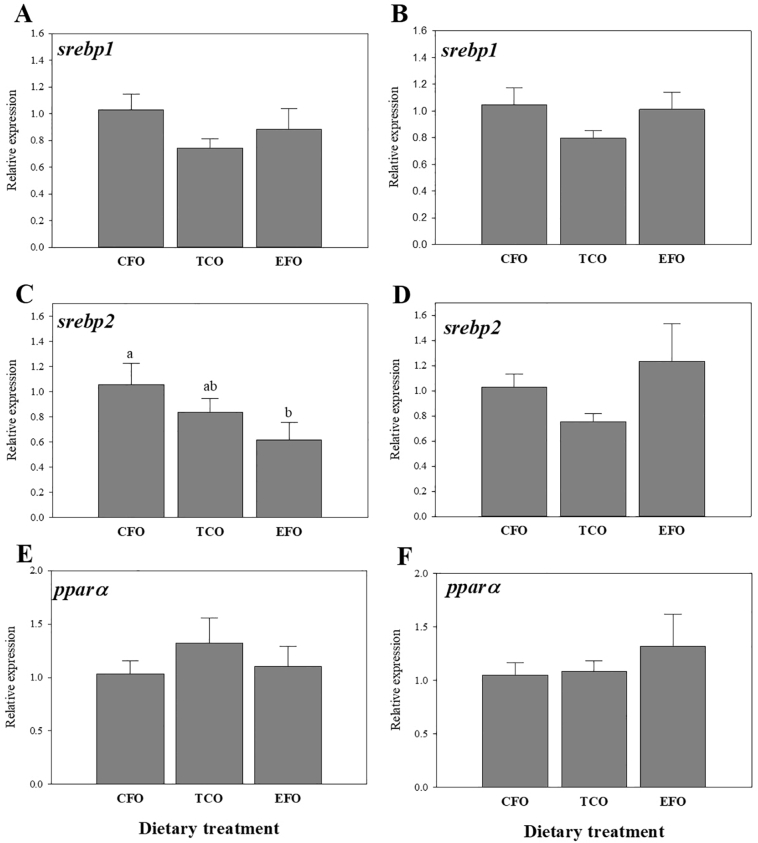
Expression of transcription factor genes in liver (A, C and E) and anterior intestine (B, D and F) of European seabass as determined by qPCR. Results are normalized expression ratios (mean ± standard error; n = 6). Different superscript letters denote differences in expression as determined by one-way ANOVA (*p* < .05). Diets contained either commercial fish oil (CFO), transgenic *Camelina sativa* oil (TCO), or fish oil at an enhanced DHA and EPA level (EFO). *Ppar*α, peroxisome proliferator-activated receptor *α*; *srebp1*, sterol regulatory element binding protein 1; *srebp2*, sterol regulatory element binding protein 2.

As observed with *fas*, although there were no significant differences in expression of sterol regulatory element binding protein 1 (*srebp1*) in liver (*p* = .256; Fig. 4A) or anterior intestine (*p* = .256; Fig. 4B), expression was numerically lowest in fish fed TCO in both tissues. This pattern was also observed for expression of *srebp2* in anterior intestine (Fig. 4D), but expression of *srebp2* in liver was significantly lower in fish fed EFO compared to fish fed CFO, with expression level intermediate in fish fed TCO tissue (Fig. 4C). There was no significant dietary regulation of peroxisome proliferator active receptor α (*pparα*) in either tissue, although highest expression in liver was observed in fish fed diet TCO (Fig. 4E), whereas expression in anterior intestine was numerically highest in fish fed diet EFO (Fig. 4F).

## 4 Discussion

Sustainable growth of aquaculture requires new, scalable sources of DHA and EPA that can improve the declining n-3 LC-PUFA content of commercial feeds and, at the same time, do not compromise growth or feed efficiency. Genetic modification of oilseed crops can provide viable and feasible new sources of EPA and DHA for fish feeds to supplement the finite supply of FO (Tocher et al., 2019). In the present study, the impacts of an oil derived from GM-*Camelina sativa* plants containing both EPA and DHA on growth performance, feed efficiency, fatty acid profile and metabolic gene expression was evaluated in European sea bass for first time. In particular, a diet containing the *de novo* oil (TCO) was benchmarked against a commercial feed formulation (CFO) and a feed formulated with conventional ingredients and enhanced levels of EPA and DHA from fish oil (EFO). Results were promising, showing that the inclusion of TCO did not impact on any of the performance parameters evaluated, confirming that this oil is an effective substitute for FO in Mediterranean fish species, as previously reported for gilthead sea bream (Betancor et al., 2016b).

Previous studies showed that one of the main adverse effects of the complete (100%) substitution of FO by VO in carnivorous marine species such as sea bass was reduced growth and general performance of the fish (Geay et al., 2010, Geay et al., 2015; Torrecillas et al., 2016, Torrecillas et al., 2017; Mu et al., 2018). While there were no significant differences in final weight across dietary treatments in the present study, SGR was significantly higher in fish fed the enhanced EFO diet compared to fish fed TCO. The reduction in SGR was positively related with feed intake (FI), with fish fed EFO and TCO showing the highest and lowest FI, respectively, with FI intermediate in fish fed CFO. Similarly, sea bream fed diets containing another GM-derived oil with high EPA displayed slightly lower SGR and FI than fish fed a diet containing FO or a GM-derived oil with high EPA + DHA similar to the one used in the present trial (Betancor et al., 2016b). However, it must be noted that in the aforementioned trial, feeds contained high levels of FM (>48%), whereas in the present trial the diets were formulated to reflect the present standard in commercial feeds with reduced FM (25%) that, in turn, could explain the differences between the impact of the GM-derived oil with high EPA + DHA between the two species. Several studies have shown previously that diets lacking or with low levels of FM and/or FO may have an adverse impact on palatability of the feed and so initially impair FI (Deng et al., 2006; Fortes da Silva et al., 2010; Luz et al., 2018). Indeed, FI and SGR were only lower in fish fed TCO in the first month of the study and after that, there were no significant differences. What was also clear in the present trial is that the initial differences in FI, potentially due to reduced palatability, can be overcome as demonstrated by the lack of difference in FI for the overall trial among fish fed the different dietary treatments. In agreement, a study investigating voluntary food intake demonstrated a greater preference for FO-based diets compared to VO diets in juvenile rainbow trout but largely at the start of the trial (Geurden et al., 2005). Additionally, Brassicaceae, such as *Camelina sativa*, are known to be contain glucosinolates (Berhow et al., 2013), which are responsible for the bitter or sharp taste of many cruciferous vegetables (Clarke, 2010). In this respect, studies with ingredients rich in glucosinolates have shown reduced growth due to negative palatability (Francis et al., 2001), which may support the observation of lower SGR in fish fed the TCO diet due likely to initially lower palatability of that feed. However, FI in fish fed the TCO diet surpassed numerically FI in fish fed the CFO diet at the end of the trial (40.2 ± 1.9 and 39.8 ± 1.2 g tank^−1^, respectively), suggesting that issues of negative palatability were transient and could potentially be avoided if glucosinolate level was reduced or specific attractants were added to the feed. The biotechnological reduction of glucosinolates in *C. sativa* is a logical and tractable target for future manipulation (Nour-Eldin et al., 2017).

As expected, the tissue fatty acid profiles mirrored those of the diets, at the same time reflecting the specific metabolic/physiological functions of each tissue. In this sense, the fatty acid composition of the brain was the most conserved and less affected by diet, with the highest DHA content (16.3% on average). The relevance that DHA has for neural function and that DHA-deficient diets can lead to visual and behavioural alterations is widely known (Bell and Tocher, 1989; Benítez-Santana et al., 2012), explaining why DHA is selectively retained in brain to maintain vital neural functions. Indeed, the conserved fatty acid composition of brain in the present trial was in agreement with previous studies in different anadromous and marine fish species (Brodtkorb et al., 1997; Bell et al., 1999; Benedito-Palos et al., 2010; Betancor et al., 2014, Betancor et al., 2016b; Rombenso et al., 2016), indicating the robustness of this tissue to dietary changes in lipid source. A common aspect in all the evaluated tissues, was the presence of significantly higher levels of ARA in fish fed TCO. ARA is an essential fatty acid for fish and inadequate levels can impact on fish growth and performance (Norambuena et al., 2015; Torrecillas et al., 2018; Chee et al., 2020). Additionally, ARA plays a key role in initiating inflammatory responses both in humans (Tallima and El Ridi, 2018) and fish (Li et al., 2012; Dantagnan et al., 2017), mediated through their potent bioactive eicosanoid products (Calder, 2009). Nonetheless, other C_20_ PUFA, such as EPA, are known to modulate eicosanoid metabolism by both inhibiting the conversion of ARA to eicosanoids as well as by being converted to eicosanoids with different, often milder, activity compared to their ARA homologues (Weber, 1990). In the present trial, the higher tissue levels of ARA in TCO-fed fish were compensated by increased deposition of both EPA and 20:4n-3. Indeed, all the tissues of fish fed TCO displayed increased levels of the latter fatty acid, and it is known that supplementation of rainbow trout macrophages with 20:4n-3 decreased the conversion of ARA into prostaglandins, while 20:4n-3 was simultaneously converted into eicosanoids with ARA-antagonising properties (Samel et al., 1987; Ghioni et al., 2004). In the present trial, the level of 20:4n-3 was at least double in fish fed TCO than in those of the other treatments, with a 6-fold increase in the case of liver, which could potentially compensate for the higher ARA levels, maintaining a balanced production of pro- and anti-inflammatory lipid mediators.

Inclusion of the oil derived from the GM oilseed crop promoted the deposition of the health beneficial fatty acids EPA and DHA in the flesh of sea bass at levels comparable to fish fed the commercial standard (CFO) and n-3 LC-PUFA enhanced (EFO) diets that were formulated with fish oil. Sea bass is regarded as a lean fish, accumulating low levels of fat in their muscle, as observed in the present trial (~4% on average) and, as expected, closely mirroring the fatty acid profile of the diets (Betancor et al., 2016b; Bordignon et al., 2020). In addition, the flesh of fish fed TCO displayed levels of n-3 docosapentaenoic acid (DPA; 22:5n-3) > 2-fold higher than those fed the other two diets. The n-3 DPA may play a role in imparting the health benefits previously attributed solely to EPA and DHA (Drouin et al., 2019) and thus the higher level of this fatty acid in flesh of fish fed TCO might offer enhanced health benefits compared to fish fed the other diets. Of course, it must be acknowledged that the experimental fish were not of harvest size and that lipid and fatty acid deposition may vary with fish size as has been demonstrated previously (Salini et al., 2016; Ren et al., 2017). Red blood cells (RBC) have been acknowledged as a good proxy for flesh fatty acid profile, at least in Atlantic salmon (Schlechtriem et al., 2009). In the present study, the fatty acid compositions of RBC and flesh were generally similar, showing the same patterns in both n-6 and n-3 LC-PUFA, with similar levels of DHA among fish fed the different dietary treatments. In agreement, studies have found that DHA levels in RBC were relatively stable, even when fish were fed diets poor in n-3 LC-PUFA (Waagbø et al., 1993, Waagbø et al., 1995; Montero et al., 2003).

In contrast, liver and anterior intestine are known to be two of the most active metabolic tissues in fish (Tocher, 2003; Betancor et al., 2016a). This can be corroborated in view of the present results, as both tissues presented differences in the levels of EPA and DHA among fish fed the different diets, with fish fed TCO exhibiting the highest levels in the case of liver, whereas fish fed TCO and EFO showed similar levels of DHA in the intestine. The expression of genes of LC-PUFA biosynthesis in these target tissues in part supported this hypothesis, showing up-regulation of *elovl5* expression in liver and, particularly, anterior intestine of fish fed TCO. In contrast, the relative expression levels of *fads2* were similar among fish fed all the dietary treatments. Although this desaturase is generally considered to be the rate-limiting step in the LC-PUFA biosynthesis pathway (Vagner and Santigosa, 2011), it is known that *fads2* is barely functional in seabass (González-Rovira et al., 2009), which might explain the lack of nutritional regulation. However, it is important to note that, in the present study, there is no negative control diet with low LC-PUFA levels (*e.g.* VO alone) and that the dietary levels of EPA + DHA in all feeds were well above requirements. This also might explain why no differences were observed in the expression of *fads2* in contrast to results obtained with other marine species where this gene was up-regulated in fish fed low levels of LC-PUFA (Izquierdo et al., 2008; Betancor et al., 2016b; Houston et al., 2017; Jin et al., 2017). Surprisingly, another tissue that did not entirely reflect dietary fatty acid profiles was gill, where fish fed TCO exhibited the highest total n-3 LC-PUFA levels. Gills constitute one of the first physical barrier defences of fish, playing a pivotal role in osmoregulation (Bell et al., 1996), and dietary interventions often lead to alteration of the fatty acid profile. In a previous study in sea bream, some indication of LC-PUFA biosynthesis was observed in fish fed a GM-derived Camelina oil with high EPA level (Betancor et al., 2016b), and another study demonstrated dietary regulation of *fads2d5* in Atlantic salmon gills, particularly in response to ARA and EPA levels (Betancor et al., 2014). Therefore, in future studies, it would be interesting to evaluate the impact that feeds containing these *de novo* oils have, not only on gill composition, but also on their molecular response.

Gene expression in the target tissues liver and anterior intestine was used as an indicator of metabolic effects of diet with the focus on lipid and energy metabolism and their transcriptional regulation. Modest dietary regulation in these pathways was observed in liver, but no significant differences were detected in intestine other than for *elovl5*. Regarding transcription factors, liver of fish fed the standard commercial diet showed up-regulation of *srebp-2*, whose activity is controlled by cellular cholesterol status (Horton et al., 2003). Indeed, Srebp-2 regulates the expression of genes involved in cholesterol synthesis (Jeon and Osborne, 2012; Carmona-Antoñanzas et al., 2014) and is up-regulated in response to reduced cholesterol in fish (Minghetti et al., 2011; Carmona-Antoñanzas et al., 2014). However, the expression levels of *srebp2* in liver in the present study did not reflect the level of dietary FO, which, in turn, is a proxy for dietary cholesterol as FO contains cholesterol, whereas VO do not (Gunstone and Harwood, 2007). It should be noted, however, that on top of being devoid of cholesterol, VO can be rich in phytosterols that can reduce plasma cholesterol in mammals (Moreau et al., 2002), although this may not be the case in fish (Liland et al., 2013; van Spankeren et al., 2019). Regardless, *srebp2* was up-regulated in salmon when fed diets with a high phytosterol:cholesterol ratio (Liland et al., 2013). Although dietary sterol levels were not determined in the present trial, it can be speculated that the rapeseed oil used in the formulation of CFO, contained higher levels of phytosterols, that in turn lead to the up-regulation of *srebp2*, because rapeseed oil is known to contain relatively high levels of phytosterols, compared to other commonly used VO (Yang et al., 2019).

On the other hand, there was increased expression of the lipid catabolic gene *cpt1a* in sea bass fed EFO compared to CFO, while TCO-fed fish displayed intermediate values. Up-regulation of *cpt1* is a common finding when fish are fed high dietary lipid or n-3 LC-PUFA (Peng et al., 2014; Xiao et al., 2017), which was in agreement with the present study, as levels of dietary n-3 LC-PUFA were varied between CFO, and the TCO and EFO. Another gene in the liver regulated by diet was the fatty acid transporter, *fabp1*, which was up-regulated in fish fed EFO. Previous studies have described a correlation between β-oxidation capacity and expression levels of *fabps* in fish (Londraville and Sidell, 1996; Torstensen et al., 2009), which was consistent with the results of the present study as indicated by the expression patterns of *cpt1a* and *fabp1* in liver.

The present study has contributed new information supporting the efficacy of GM Camelina oil as a replacement for FO in feeds for European seabass. Fish fed the GM-derived oil grew equally to fish fed either a standard commercial diet or one with enhanced levels of EPA + DHA. Slightly reduced growth of fish fed TCO at the beginning of the trial was related to lower feed intake, possibly caused by glucosinolates in *Camelina sativa*, but was transient and disappeared after the first month of feeding. The GM-derived oil improved the nutritional quality of the fish fillet, enhancing total n-3 PUFA level compared to fish fed the other two diets. The small changes in dietary LC-PUFA did not trigger a strong and consistent response from a molecular point of view, while there were indications of endogenous production of LC-PUFA in tissues such as liver, intestine and gill. The high levels of LC-PUFA present in TCO and EFO appeared to trigger a metabolic response consisting of an up-regulation of both β-oxidation (*cpt1a*) and fatty acid transport (*fabp1*). The present study corroborates that oils derived from GM crops are an excellent source of EPA and DHA and thus a substitute for FO in diets for marine carnivorous species, helping to fill the gap between supply and demand for n-3 LC-PUFA.

The following is the supplementary data related to this article.

Supplementary Table S1 Primers used in qPCR analysis. Supplementary Table S1

## Declaration of Competing Interest

The authors declare no conflict of interest exist.
