# Supplementary material for: Oil from transgenic Camelina sativa as a source of EPA and DHA in feed for European sea bass (Dicentrarchus labrax L.)
Source: Aquaculture. 2021 Jan 15;530:735759. doi: 10.1016/j.aquaculture.2020.735759 (PMC7729833; doi:10.1016/j.aquaculture.2020.735759)
Supplement: Supplementary Table S1 — Primers used in qPCR analysis. [file mmc1.docx]

**Supplementary Table 1**. Primers used in qPCR analysis.

|  |  |  |  |  |  |  |
| --- | --- | --- | --- | --- | --- | --- |
| Transcript | Primer sequence (5'→3') | | Amplicon (bp) | Accession no. | |  |
| *cpt1a* | F: AGGCTAGGTGAGATTCGGGT | | 204 | KF857302.1 | |  |
|  | R: ACTGGACAATCCTTCGGCTG | |  |  | |  |
| *elovl5* | F: GCCGTACCTTTGGTGGAAGA | | 234 | FR717358.1 | |  |
|  | R: GATACGGGAGAGCCGTTCTG | |  |  | |  |
| *fabp1* | F: CCGGCTCAAAGGTCCTCATT | | 223 | FL486710.1 | |  |
|  | R: TACATGCGTTTGCTCGTCCT | |  |  | |  |
| *fads2* | F: TGTATGGCCTGTTTGGCTCG | | 152 | EU439924.1 | |  |
|  | R: GGTGGCTTGTAACTGCATGG | |  |  | |  |
| *fas* | F: CGTCAAGCTCTCCATCCCTG | | 238 | MF566098 | |  |
|  | R: GGTGGTGTCTAGGCAGTGTC | |  |  | |  |
| *ppara* | F: ATGGTGACTATCCGGAGCCCG | | 216 | AY590300 | |  |
|  | R: ACTTCAAGAGAGTCACCTGGTCAT | |  |  | |  |
| *srebp1* | F: CACTCCAAGTGGTGGTCCTC | | 189 | FN677951.1 | |  |
|  | R: TTCTGGCTTGGACAGCAGAG | |  |  | |  |
| *srebp2* | F: CGTGTGACTTGGGAACCAGA | | 192 | MF438039 | |  |
|  | R: AAGAGTGAGCCAGATTCGCC | |  |  | |  |
| *bactin* | F: GTGGCTACTCCTTCACCACC | | 81 | AJ537421.1 | |  |
|  | R: CGGAACCTCTCATTGCCGAT | |  |  | |  |
| *ef1a* | F: GGAGTGAAGCAGCTCATCGT | | 199 | AJ866727.1 | |  |
|  | R: AGCCCATCTTTACACTGCCC | |  |  | |  |
| *rplp0* | F: CAGGCTCTGGGTATCACCAC | | 197 | FM018449.1 | |  |
|  | R: AGCACCTCAGGGCTGTAAAC | |  |  | |  |
|  |  |  | | |  | |

*cpt1a*, carnitine palmitoyltransferase 1A liver isoform; *elovl5*, fatty acid elongase 5; *fapb1*, fatty acid binding protein 1; *fads2*, fatty acid desaturase 2; *fas*, fatty acid synthase; *pparα*, peroxisome proliferator-activated receptor α; *srebp1*, sterol regulatory element binding protein 1; *srebp2*, sterol regulatory element binding protein 2; *bactin*, beta actin; *ef1*α, elongation factor 1; *rplp0*, ribosomal protein lateral stalk subunit P0
